# *Streptomyces flavogriseus* HS1: Isolation and Characterization of Extracellular Proteases and Their Compatibility with Laundry Detergents

**DOI:** 10.1155/2014/345980

**Published:** 2014-04-06

**Authors:** Sofiane Ghorbel, Maher Kammoun, Hala Soltana, Moncef Nasri, Noomen Hmidet

**Affiliations:** Laboratoire de Génie Enzymatique et de Microbiologie, Ecole Nationale d'Ingénieurs de Sfax, BP 1173, 3038 Sfax, Tunisia

## Abstract

The present study describes the isolation of a new protease producing *Streptomyces* strain HS1 and the biochemical characterization of the secreted proteases. By sequencing of its noted 16S rDNA, HS1 strain was found to have a 100% identity with *Streptomyces flavogriseus*. The highest protease production was found using FermII media. In these conditions maximum protease production (99 U/mL) was obtained after 96 h incubation at 30°C and 150 rpm. HS1 strain produced at least five proteases as revealed by zymogram technique. The enzyme preparation exhibited activity over a broad range of pH (5–11) and temperature (25–70°C). Optimum activity was observed at a pH of 7.0 and a temperature of 50°C. Proteolytic activity was significantly unaffected by Ca^2+^ and Mg^2+^. EDTA and PMSF highly decreased the original activity. The crude extracellular proteases showed high stability when used as a detergent additive. These properties offer an interesting potential for enzymatic hydrolysis at the industrial level.

## 1. Introduction

Actinomycetes, a Gram-positive filamentous bacteria, can degrade various macromolecules in soil [1]. Among actinomycetes,* Streptomyces* species are the most industrially useful because of their capacity of producing numerous secondary metabolites, particularly antibiotics. Similarly, these bacteria offer a second industrial interesting use by producing large amounts of proteolytic enzymes, with different substrate specificities [2]. The investigation of proteases does not take place only in scientific fields such as protein chemistry and protein engineering, but also in other industrial uses including cleaning detergents, leather, and food additives. Such a wide use of proteases in the industrial field shows their importance especially that they represent around 60% of the total enzyme market [3]. Unlike proteases from other bacteria which have been extensively characterized, proteases from actinomycetes did not receive a similar attention [4]. Still, their ability to produce a variety of enzymes may be an attractive phenomenon of these prokaryotes.

The composition of the protease complexes secreted by* Streptomyces* is determined by the taxonomic position of the producers [5–8]. The potential use of* Streptomyces* for producing proteases is justified by their ability to release the proteins into extracellular media. Such a capability is generally regarded as safe (GRAS) with food and drug administration.* Streptomyces* spp. that produce proteases include* S. clavuligerus, S. griseus, S. rimouses, S. thermoviolaceus*, and* S. thermovulgaris* [7]. Some of these proteases, like the serine proteases of* Streptomyces griseus* [8, 9] and* Streptomyces fradiae* [10], have been characterized structurally and enzymatically. There have also been many descriptions of isolation and partial characterization of alkaline protease activities from other members of the genus* Streptomyces* like* Streptomyces clavuligerus*,* Streptomyces gulbargensis*,* Streptomyces viridifaceins*, and* Streptomyces* sp. [11–13].

Present study describes the isolation of a multiple protease producing* Streptomyces flavogriseus* HS1 strain, isolated from a Tunisian soil. We report the biochemical characterization of the crude enzyme for evaluation of its biotechnological potential as detergent additive.

## 2. Material and Methods

### 2.1. Isolation of the Actinomycete Strain

The isolation of the Actinomycete strains from a soil sample was done by serial dilution plate technique on ISP4 agar media containing (g/L) starch 10, casein 0.3, KNO_3_ 2, NaCl 2, K_2_HPO_4_ 2, MgSO_4_·7H_2_O 0.05, CaCO_3_ 0.02, FeSO_4_·7H_2_O 0.01, and 15 agar [14].

### 2.2. Identification of HS1 Strain

The 16S rRNA gene of the HS1 strain was amplified by PCR using the following primers: F (forward), 5′-CCGAATTCGTCGACAACAGAGTTTGATCCTGGCTCAG-3′ and R (reverse), 5′-CCCGGGATCCAAGCTTAAGGAGGTGATCCAGCC-3′. The PCR mixture contained 30 pmol of primers, 20 pmol of each deoxynucleoside triphosphate, polymerisation buffer, and 5 U Taq polymerase. The PCR program involved 35 cycles of denaturing at 94°C for 1 min, primer annealing at 55°C for 1 min, and extension at 72°C for 90 s. The sequencing was performed three times using the DNA sequencer ABI PRISM 3100/3100-Avant Genetic Analyser (CA, USA). 16S rDNA sequence was searched for similarities to known sequences in the GenBank database (National Center for Biotechnology Information, National Library of Medicine) using the BLAST search program. The sequence was aligned with those of the reference strains using ClustalW [15]. A phylogenetic tree was constructed by the neighbour-joining method [16].

### 2.3. Determination of Protease Activity

Measuring of the protease activity was done as described by Kembhavi et al. [17], using casein as substrate 1% (w/v) in 100 mM Tris-HCl buffer, pH 7.0. The mixture was incubated for 15 min at 50°C and the reaction was stopped by addition of 0.5 mL 20% (w/v) TCA (trichloroacetic acid). The mixture was left at room temperature for 10 minutes and then centrifuged at 10000 g for 15 minutes to remove the precipitate. The absorbance of the soluble TCA peptides was recorded at 280 nm. One unit of protease activity was defined as the amount of enzyme required to liberate 1 *μ*g of tyrosine per minute under the experimental conditions used. All measurements were carried out in triplicate.

### 2.4. Protease Production

The proteolytic isolates were cultured in three different liquid medium: FermII media: (g/L) dextrin 20, tryptone 10, KH_2_PO_4_ 1.0, K_2_HPO_4_ 3.4, MgSO_4_·7H_2_O 0.3, FeSO_4_·7H_2_O 0.01, ZnCl_2_ 0.1, CuSO_4_·7H_2_O 0.01, MgCl_2_·4H_2_O 0.003, CaCl_2_ 0.01, NaCl 0.03, pH 7.0 [18], gelatin containing media: (g/L): gelatin, 10; peptone, 5; yeast extract, 5; NaCl, 50; and pH 9, and liquid ISP4 media [14].

A 1 mL spore suspension (10^4^ to 10^6^ spores/mL) was added to a 250 mL Erlenmeyer flask containing 100 mL of liquid media [14], and the flasks were incubated at 30°C and 150 rpm for 96 h. The culture medium was centrifuged at 5000 rpm to remove mycelia and medium debris, and the supernatant was used as a crude enzyme preparation.

### 2.5. Characterization of Proteases

Zymography was performed on NativePAGE according to the method of Garcia-Carreno et al. [19]. After electrophoresis, the gel was submerged in 1% (w/v) casein in 100 mM glycine-NaOH buffer, pH 7.0, and incubated at 50°C for 20 min. After washing, the gel was stained with Coomassie Brilliant Blue R-250 and destained with 5% ethanol-7.5% acetic acid. A clear zone appeared on the blue background of the gel which indicated the presence of protease activity.

### 2.6. Effect of Temperature and pH on Protease Activity and Stability

To investigate the temperature effect, the protease assay was performed at different temperatures between 20 and 80°C, using casein as substrate for 10 min at pH 7.0. For thermal stability, the enzyme was incubated at different temperatures for 60 min. Aliquots were withdrawn at the designed time intervals to test the remaining activity. The residual activity was assayed at pH 7.0 and 50°C for 10 min. The nonheated enzyme was considered as control (100% activity).

Protease activity was assayed over the pH range 5.0–12.0 at 50°C for 10 min, using casein as a substrate. The effect of pH on enzyme stability was evaluated by measuring the residual enzyme activity after incubation at various pH for 60 min at 25°C. The following buffer systems were used: 100 mM glycine-HCl, pH 4.0 and 5.0; 100 mM sodium acetate, pH 6.0; 100 mM phosphate-buffer, pH 7.0; 100 mM Tris-HCl, pH 8.0; 100 mM glycine-NaOH, pH 9.0 and 10; and 100 mM Na_2_HPO_4_·NaOH, pH 12.0.

### 2.7. Effect of Metal Ions and Other Chemicals on Protease Activity and Stability

The effects of different monovalent (Na^+^ or K^+^) or divalent (Fe^2+^, Mn^2+^, Zn^2+^, Cu^2+^, Ba^2+^, Mg^2+^, or Hg^2+^) metal ions, at a concentration of 5 mM, on protease activity were investigated by adding them to the reaction mixture. The activity in the absence of any additives was taken as 100%.

The effects of enzyme inhibitors on protease activity were studied using PMSF and EDTA. The crude enzyme was preincubated with each inhibitor for 30 min at 25°C, and then the remaining enzyme activity was estimated using casein as a substrate. The activity in the absence of inhibitors was taken as 100%. The effects of some surfactants (Triton X-100, Tween 80, and SDS) and oxidizing agents (sodium perborate) on enzyme stability were studied by preincubating the crude enzyme for 1 h at 25°C. The residual activity was measured at pH 7.0 and 50°C. The activity of the enzyme without any additive was taken as 100%.

### 2.8. Detergent Compatibility

The compatibility of the HS1 extracellular proteases with commercial laundry detergents was studied using Ariel (Procter and Gamble, Switzerland), Newdet (Sodet, Tunisia), and Dixan (Henkel, Spain) as solid detergents. The endogenous proteases contained in these detergents were inactivated by heating the diluted detergents for 1 h at 65°C prior to the addition of the enzyme preparation. The extracellular proteases of* Streptomyces flavogriseus* HS1 were incubated with different diluted detergents (1/100) for 1 h at 30°C, 40°C, and 50°C and then the remaining activities were determined under the standard assay conditions. The enzyme activity of a control, without detergent, incubated under similar conditions, was taken as 100%.

### 2.9. Statistical Analysis

Statistical analyses were performed with Statgraphics ver. 5.1, professional edition (Manugistics Corp., USA) using ANOVA analysis. Differences were considered significant at *P* < 0.05. Results represent the means of at least two determination carried out in duplicate. The difference between values did not exceed 5%.

## 3. Results and Discussion

### 3.1. Isolation of the Actinomycete Strain

**Figure 1:**
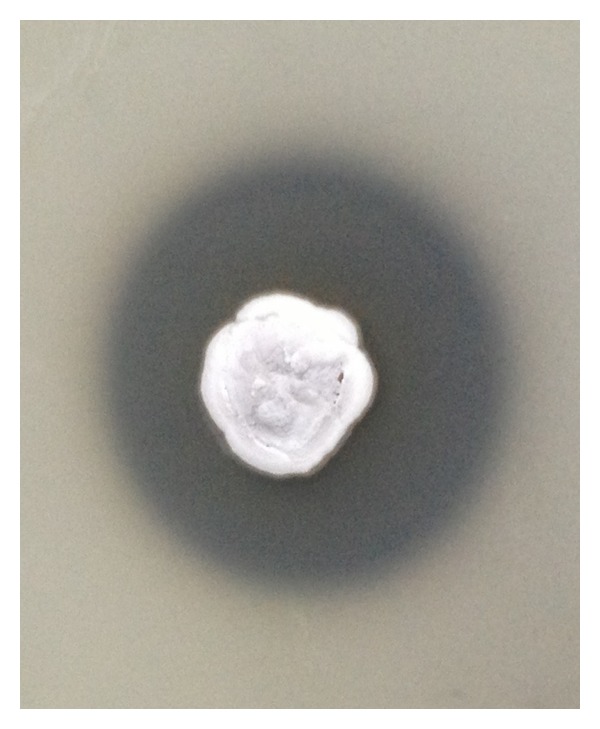
Plate assay showing the zone of proteolytic activity by protease-producing* Streptomyces flavogriseus* HS1 strain.

**Figure 2:**
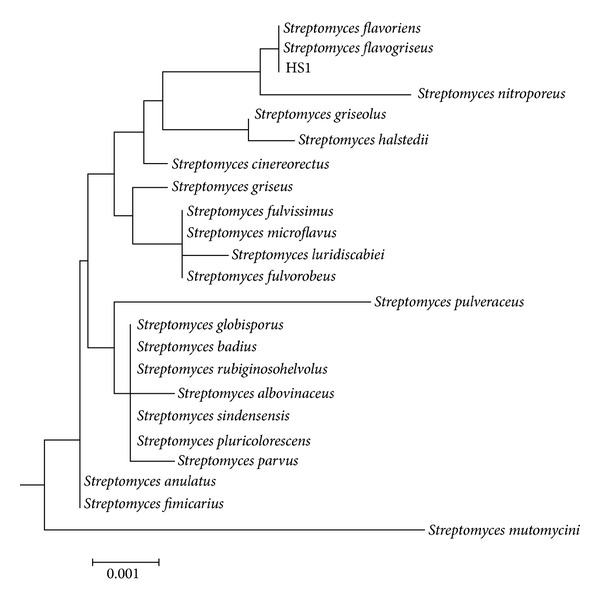
Dendrogram showing the relationships between* Streptomyces flavogriseus* HS1 and other* Streptomyces *species. Topology was inferred using the neighbour-joining method.

Samples were taken from an organic rich soil in Sfax city (Tunisia). Isolation of the actinomycete strains was obtained after 96 h of incubation at 30°C. One isolate was selected for further studies because of its important extracellular proteases secretion and named HS1 strain (Figure 1). HS1 strain was confirmed as belonging to the genus* Streptomyces* since it possessed nonfragmented substrate mycelia, aerial hyphae, and smooth spores organized in straight chains. Analysis of the 16S rRNA gene sequence of this strain showed a high similarity (100%) with* Streptomyces flavogriseus* (Figure 2).

*Streptomyces flavogriseus* was well known to produce several enzymes such as cellulose, xylanase, and glucose isomerase [19–21], but no data was found describing extracellular proteases.

### 3.2. Protease Production

Three liquid culture media optimized for the production of extracellular proteases in* Streptomyces* were tested, among them are FermII [18], ISP4 [14], and gelatin based media (this study). In the light of this experiment, FermII was found to be the best medium for the production of* Streptomyces flavogriseus* HS1 extracellular proteases (Table 1). The fermentation time course for protease production by* Streptomyces flavogriseus* HS1 (data not shown) indicates that the maximum protease activity (99 U/mL) was obtained after 96 h of cultivation, when cells were in the stationary phase, and its production was not growth associated. Similar results were obtained by Gibb and Strohl [22], who observed that the maximum protease production by* Streptomyces peucetius* occurred after 100 h of cultivation at the stationary phase growth. This period was shorter than that of the well-studied* Streptomyces* (e.g.,* Streptomyces moderatus* required 120 h of cultivation for maximum protease production [6]). However, Dastager et al. [13] showed that the protease activity measured in the cell-free supernatant fluid of* Streptomyces gulbargensis* sp. Nov. was maximum (121.8 U/mL) after 48 h of growth.

### 3.3. Zymogram

**Figure 3:**
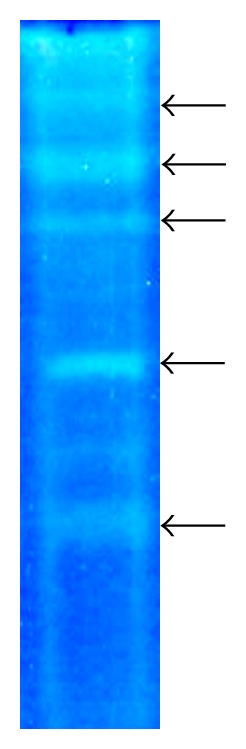
Zymography analysis of* Streptomyces flavogriseus* HS1 crude extracellular proteases.

To give more information about the diversity of extracellular proteases secreted by HS1 strain, zymogram analysis was done as described in the “Material and Methods” section. As shown in Figure 3, proteolytic activity profiles of cell-free enzymatic preparation of* Streptomyces flavogriseus* HS1 showed at least five major proteases. This is a common feature for the streptomycetes [6, 23]. However, the nature and characteristics of the enzymes of protease complex derived from streptomycetes have not been widely studied [6]. All the thermophilic bacterial extracellular proteases so far reported are, interestingly, serine or neutral metalloproteases [24].

### 3.4. Effect of the pH on Activity and Stability of* Streptomyces flavogriseus* HS1 Extracellular Proteases

**Figure 4:**
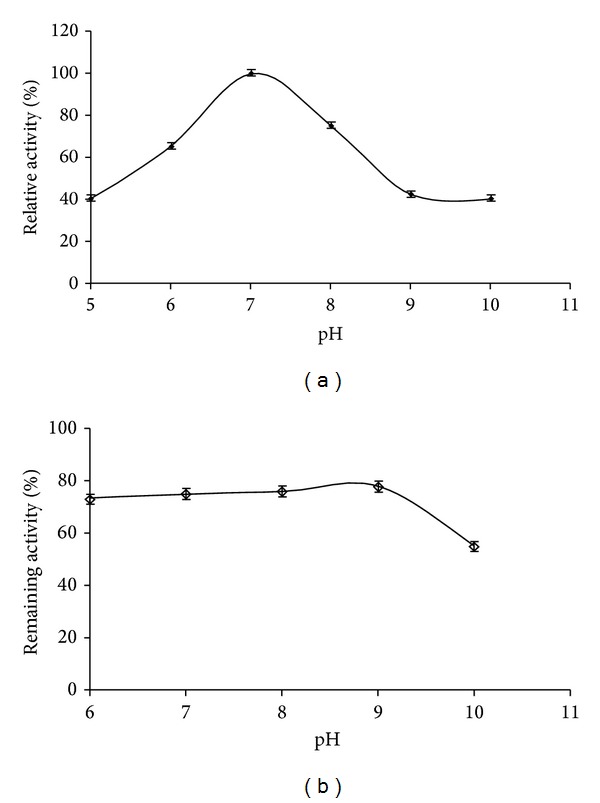
Effect of pH on activity (a) and stability (b) of the crude protease from* Streptomyces flavogriseus* HS1. The protease activity was assayed in the pH range of 5.0–12.0 using buffers of different pH values at 50°C. The maximum activity obtained from pH 7.0 was considered as 100% activity. The pH stability of the* Streptomyces flavogriseus* HS1 crude enzyme was assayed in the rage of 5.0–12.0 and determined by incubating the crude protease in different buffers for 1 h at 25°C and the residual activity was determined at pH 7.0 and 50°C. The proteolytic activity before incubation was taken as 100%.

The pH profile of protease activity from* Streptomyces flavogriseus* HS1 is shown in Figure 4(a). The crude enzyme was highly active between pH 6.0 and 8.0, having an optimum around pH 7.0. The relative activities at pH 6.0 and 8.0 were about 65 to 75%. Similar results were described for several* Streptomyces* strains in the literature with optimum pH range being between 6.0 and 12.0 [25]. The optimum pH activity of* Streptomyces flavogriseus* proteases was similar to that from other* Streptomyces* species, such as* Streptomyces griseus* pronase [26]. Therefore, this activity was lower than that of other* Streptomyces* described proteases, showing maximum activity at pH 8.0 like* Streptomyces* sp. DP2 [27] and* Streptomyces* sp. CN902 [28]. The pH stability test showed that the crude proteases were highly stable over a broad pH range, maintaining more than 70% of its original activity between pH 5.0 and 9.0 (Figure 4(b)).

### 3.5. Effect of Temperature on the Activity and Stability of* Streptomyces flavogriseus* HS1 Extracellular Proteases

**Figure 5:**
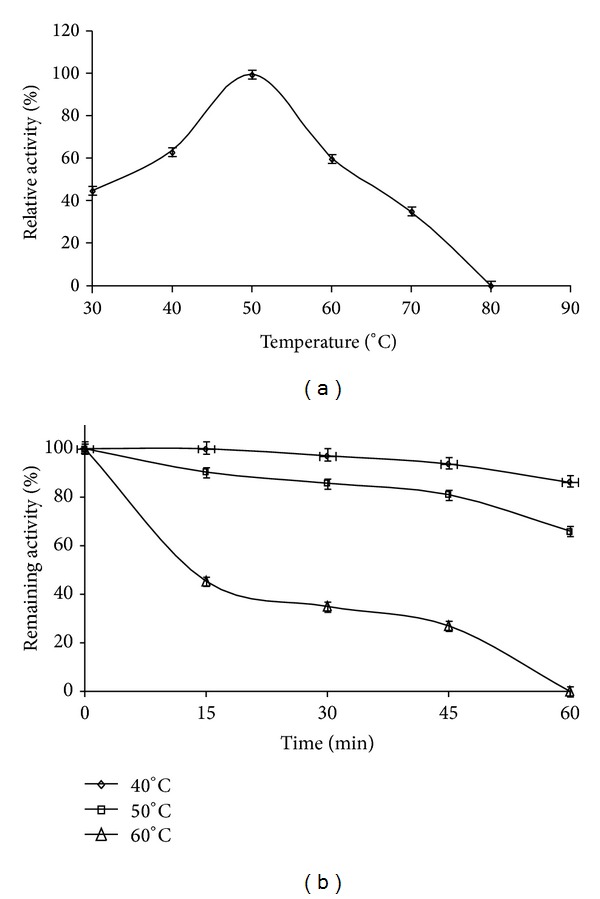
Effect of temperature on activity (a) and stability (b) of the crude protease from* Streptomyces flavogriseus* HS1. The temperature profile was determined by assaying protease activity at temperatures between 30 and 80°C. The activity of the crude enzyme at 50°C was taken as 100%. The temperature stability was determined by incubating* Streptomyces flavogriseus* HS1 crude enzyme at temperatures from 40 to 60°C for 1 h. The residual proteolytic activity was determined at regular intervals under standard assay conditions. The original activity before preincubation was taken as 100%.

The temperature profile of protease activity from* Streptomyces flavogriseus* HS1 is presented in Figure 5(a). The HS1 crude extract was active at temperatures from 30 to 70°C and had an optimum at 50°C, while activity decreased rapidly above 70°C. The relative activities at 40 and 60°C were about 63% and 60%, respectively. Several actinomycete thermophilic proteases, with high activity at 70°C, have been reported for* Thermoactinomyces vulgaris* and* Nocardiopsis dassonvillei*,* S. corchorusii* [23],* S. megasporus* [29],* S. thermovulgaris* [4], and* S. thermoviolaceus* [7]. Above 60°C, protease activity from* Streptomyces flavogriseus* HS1 rapidly fell, as shown for proteases of* Streptomyces* spp. and other bacteria.

The thermal stability profile of the crude enzyme showed a high stability at temperatures below 40°C but was inactivated at higher temperatures (Figure 5(b)). After 45 min of incubation at 60°C, 78% of the initial activity was lost.* Streptomyces flavogriseus* HS1 proteases were stable at 40 and 50°C after 1 h incubation. At low temperatures (−20 and 4°C), the crude enzyme preparation retained 75% of its activity after 2 months. El-Raheem et al. [23] observed that for alkaline proteases from a strain of* S. corchorusii*, activity did not decrease after storage at −20°C for one year, at pH values between 4.0 and 12.0, and repeated freezing and thawing.

### 3.6. Effects of Metal Ions 

The effects of some metal ions, at a concentration of 5 mM, on the activity of* Streptomyces flavogriseus* HS1 crude enzyme were studied at pH 7.0 and 50°C by the addition of the respective cations to the reaction mixture (Table 2). The Ca^2+^, Mg^2+^, and Na^+^ were shown to have no effect on the protease activity. The latter was slightly affected by Ba^2+^ and K^+^ and it retains about 79.1% and 66.6% of its activity, respectively. The Hg^2+^, Cu^2+^, and Zn^2+^ greatly affected the enzymatic activity till the total inhibition. Reduction in protease activity of* Streptomyces* spp. has been previously observed [30, 31], especially for Cu^2+^, probably as a result of the denaturing action of copper [31] and the chelating effect of EDTA. Proteases from* Streptomyces* spp. and* N. dassonvillei* were also stimulated by Mg^2+^, Mn^2+^, Ca^2+^, and Zn^2+^ [30], whereas cation-requiring proteases from streptomycetes have also been reported [32]. However,* Streptomyces* sp. 594 protease stability was enhanced only by Ca^2+^ and Ba^2+^ [33]. It was shown that the Ca^2+^ ions are important for catalysis. James et al. [7] suggested that most probably they stabilize the protein through specific or nonspecific binding sites and may also allow for additional bonding within the enzyme molecule, preventing unfolding at higher temperatures, as has been demonstrated for protease from thermophilic bacteria, mainly thermolysin.

### 3.7. Effects of Enzyme Inhibitors on Protease Activity

Proteases can be classified by their sensitivity to various inhibitors [34]. In order to confirm the nature of the extracellular proteases, the effects of different enzyme inhibitors, such as chelating agent and a specific group of reagents, on the protease activity were investigated (Table 3). The crude proteolytic preparation was strongly inhibited by the serine protease inhibitor (PMSF) indicating that the HS1 crude extract contained serine proteases. In addition, the enzymatic extract was also inhibited by the chelating agent EDTA (5 mM), with 77% of its original activity being lost, indicating the importance of ions in enzyme stabilization. These findings are in line with several earlier reports showing that active structure of serine proteases contains Ca^2+^ binding site(s) and the removal of Ca^2+^ from the strong binding site is associated with a significant reduction in thermal stability [35]. From this result, HS1 can contain serine metalloproteases since the crude enzyme is strongly inhibited by both PMSF and EDTA.

### 3.8. Effects of Oxidizing Agents and Surfactants on Protease Stability

In order to be effective during washing, a good detergent protease must be compatible and stable with all commonly used detergent compounds such as surfactants, oxidizing agents, and other additives, which might be present in the detergent formulation [29, 30]. HS1 protease extract was preincubated 60 min at 25°C in the presence of SDS, Tween 20 and 80, and Triton X-100 and the residual activities were assayed at pH 7.0 and 50°C (Table 4). Interestingly, the* Streptomyces flavogriseus* HS1 proteases were less stable against the strong anionic surfactant (SDS) and retained only 19% of its activity in the presence of 0.1% (w/v) SDS. However, HS1 protease activity was little influenced by oxidizing agents and retained 64.1% and 39.8% of its activity after incubation for 1 h at 25°C in the presence of 0.1% and 1% sodium perborate, respectively (Table 4). The stability of the enzyme in the presence of oxidizing agents is a very important characteristic for its eventual use in detergent formulations. Important commercial detergent proteases like Subtilisin Carlsberg, Subtilisin BPN′, Alcalase, Esparase, and Savirase are stable in the presence of various detergent components. However, most are unstable in the presence of oxidant agents, such as hydrogen peroxide [36].

### 3.9. Stability of the* Streptomyces flavogriseus* HS1 Proteases with Commercial Solid Detergents

**Figure 6:**
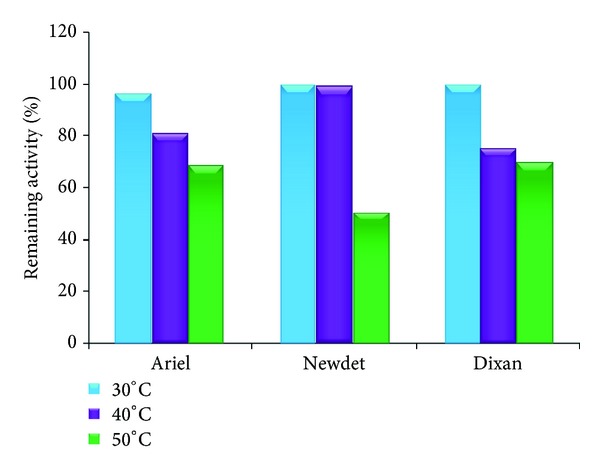
Stability of crude protease from* Streptomyces flavogriseus* HS1 in the presence of various commercial solid detergents. The enzyme at 100 U/mL was incubated 1 h at 30°C, 40°C, and 50°C in the presence of solid detergents. The remaining activities were determined at pH 7.0 and 50°C using casein as a substrate. Enzyme activity of control sample without any detergent, incubated under the similar conditions, was taken as 100%.

The high activity and stability of the* Streptomyces flavogriseus* HS1 proteases in the pH range 5.0–10.0 and their relative stability towards surfactants and oxidizing agents are very useful for its eventual application as a detergent additive. To check the compatibility of the proteases with solid detergents, the crude enzyme was preincubated in the presence of various commercial laundry solid detergents for 1 h at different temperatures (30, 40, and 50°C) (Figure 6). The data showed that the proteases were stable in Ariel and Dixan, retaining 70% of their activity at 50°C. The obtained results clearly indicated that the performance of enzymes in detergents depends on number of factors, including the detergents compounds since the proteolytic stability of HS1 proteases varied with each laundry tested detergent.

Singh et al. [37] reported that the alkaline protease from* Bacillus* sp. SSR1 retained 37% of its initial activity after 1 h incubation at 40°C in the presence of Ariel at a concentration of 5 mg/mL. Alkaline protease from* Conidiobolus coronatus* retained only 16% activity in Revel, 11.4% activity in Ariel, and 6.6% activity in Wheel at 50°C, in the presence of 25 mM CaCl_2_ [38].

## 4. Conclusion

This work describes the isolation of an actinomycete strain identified as* Streptomyces flavogriseus* HS1, which produces at least five proteases as described by zymogram technique. Crude protease was shown to have optimum activity at pH 7 and 50°C. The crude enzyme has a good stability toward the oxidizing agents and was found to be useful as a detergent additive.

## Figures and Tables

**Table 1:** Effect of various culture media on the production of extracellular proteases from *Streptomyces  flavogriseus* HS1.

Medium	Proteolytic activity (U/mL)
FermII	98
Gelatin based medium	4.7
ISP4	51.8

The activity of the proteases was determined at 50°C and pH 7.0 after 96 h of culture.

**Table 2:** Effect of various metal ions (5 mM) on the activity of crude enzyme from *Streptomyces  flavogriseus* HS1.

Ions (5 mM)	None	Ca^2+^	Mg^2+^	Fe^2+^	Mn^2+^	Cu^2+^	Ba^2+^	Hg^2+^	Na^+^	K^+^
Relative activity (%)	100	100	100	71	100	0	79	0	100	67

The activity of the proteases was determined by incubating the enzyme in the presence of various metal ions for 10 min at 50°C and pH 7.0.

**Table 3:** Effect of various enzyme inhibitors (5 mM) on the activity of extracellular proteases from *Streptomyces  flavogriseus* HS1.

Inhibitors	Remaining activity (%)
None	100
PMSF	5
EDTA	23

The secreted proteases from *Streptomyces  flavogriseus* HS1 were preincubated with various enzyme inhibitors for 30 min at room temperature and the remaining activity was determined at pH 7.0 and 50°C. Crude protease activity measured in the absence of any inhibitor was taken as 100%.

**Table 4:** Stability of the *Streptomyces  flavogriseus* HS1 extracellular proteases in the presence of various surfactants and oxidizing agents.

Detergents	Concentrations (%)	Remaining activity (%)
Triton X-100	1%	61.4
	5%	40.5

Tween 80	1%	71.4
	5%	40.5

Tween 20	1%	70.2
	5%	37.8

SDS	0.1%	18.9
	0.5%	0

Sodium perborate	0.1%	64.1
	1%	39.8

H_2_O_2_	0.1%	61.4
	0.5%	58.7

The crude extracellular proteases were incubated with different surfactants and oxidizing agents for 1 h at 25°C and the remaining activity was measured under standard conditions. The activity is expressed as a percentage of the activity level in the absence of additives.
